# Digital Disparities in Healthcare: A Tale of the Haves and Have-Nots?

**DOI:** 10.1177/23743735251343585

**Published:** 2025-05-21

**Authors:** Allen M. Chen

**Affiliations:** Department of Radiation Oncology, University of California, Irvine, Chao Family Comprehensive Cancer Center, Orange, CA, USA

**Keywords:** digital health, disparities, health policy, social determinants, inclusion

## Abstract

While the digitalization of healthcare and the advent of consumer-centered technologies have led to advancements in patient engagement, it is evident that certain, underprivileged segments of society might not be benefiting. The purpose of this review was to thus analyze the expanding data focusing on digital disparities in healthcare and was designed based on the Preferred Reporting Items for Systematic Review and Meta-Analysis Protocols (PRISMA-P) statement. First, a literature search of original, peer-reviewed publications was undertaken to identify studies pertaining to disparities in the utilization of digital technologies in healthcare using a variety of customized retrieval terms. Articles published from January 2014 to January 2024 were included. Subsequently, a total of 247 peer-reviewed studies were identified which were used to construct a framework for interpretation. The core themes could broadly be categorized into digital health portals (*N* = 74), telemedicine (*N* = 57), healthcare wearables (*N* = 49), digital intervention tools (*N* = 35), and virtual education (*N* = 32). While the potential of digital health to fundamentally transform the nature of patient-centric care is increasingly being recognized, the growing “digital divide” between the “haves” and “have-nots” with respect to nearly every facet of technology implementation raises concern regarding the perpetuation of inequities across society.

## Introduction

Digital health—defined by the World Health Organization as “the systematic application of information and communications technologies, computer science, and data support informed decision-making by individuals, the health workforce, and health systems, to strengthen resilience to disease and improve health and wellness”—has been proposed as a means of elevating the patient experience by promoting engagement and shared decision-making.^1^ These platforms offer unique and growing opportunities to connect with patients thus leading to improved access, enhanced health literacy, and ultimately, better quality outcomes. While the digitization of healthcare has empowered a good portion of society, it is important to recognize, however, that others might be at risk for being left behind. Due to the pace at which technological innovation is being introduced in healthcare, the lack of technical literacy for many patients, particularly those from underserved backgrounds and on the lower end of the socioeconomic spectrum, can pose a barrier for such activities as scheduling appointments, checking results, and/or communicating with providers—tasks that are increasingly digitized in modern healthcare. Similarly, patients who lack access to digital health tools due to factors related to geography may find themselves disadvantaged. Indeed, studies have shown that the acceptance and utilization of digital health platforms have been disproportionately higher among those from more affluent backgrounds.^2^ Conversely, underrepresented minorities have been shown to have more difficulty accessing their medical records online and are less likely to use digital communication tools.^3^ While the potential of technology to ameliorate many of the traditional barriers to healthcare access is just starting to be recognized and is increasingly being highlighted in healthcare delivery models, ensuring equal access across all segments of the population is paramount. The purpose of this study was to thus conduct a systematic review of digital disparities in healthcare.

## Methods and Materials

**Figure 1.**
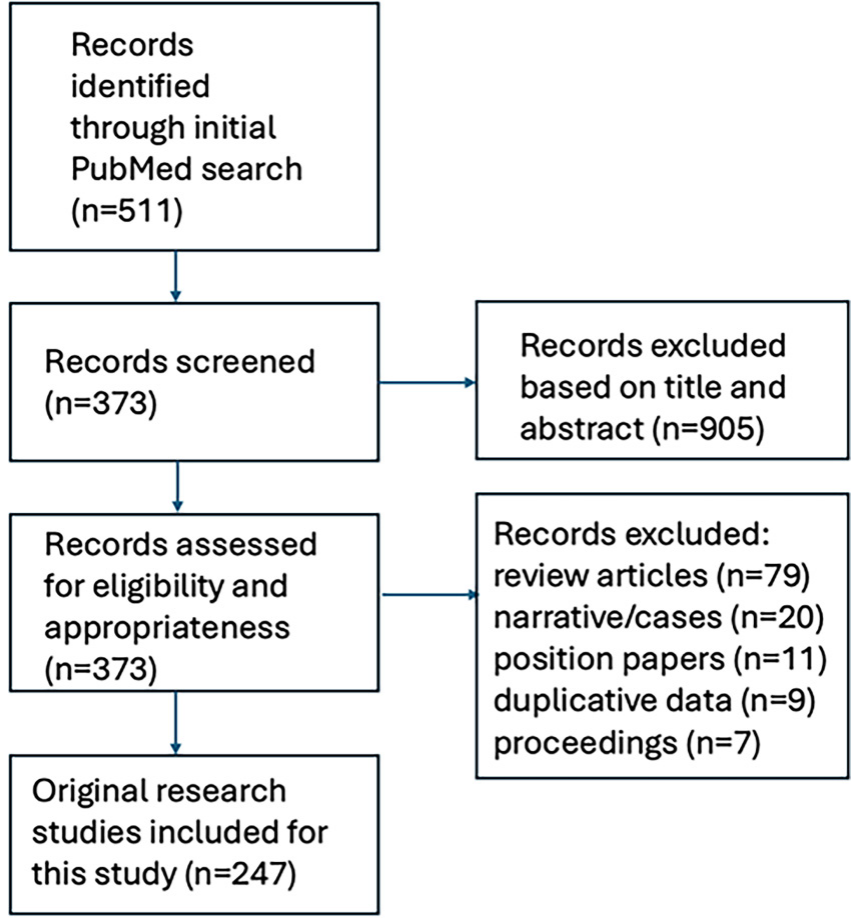
Graphical flowchart of search strategy for the systematic review.

This study was designed based on the Preferred Reporting Items for Systematic Review and Meta-Analysis Protocols (PRISMA-P) statement. A comprehensive literature search of peer-reviewed publications was undertaken to identify original peer-reviewed works pertaining to the implementation of digital health technology using a variety of search terms including “digital disparities,” “digital divide” “health technology,” “health literacy,” and “digital equity.” Search terms were inputted in various permutations to optimize yield. Both the MEDLINE and Web of Science portals were cross-referenced with one another to ensure optimization of data capture. Given the goal of critically evaluating high-level evidence which could enable the preparation of this review, the focus of this work was on specifically identifying original research reporting on the impacts of digital health on patient care. Studies were not limited to North America. The initial screen was conducted on May 1, 2024, and repeated again on June 1, 2024, July 1, 2024, and August 1, 2024. A schematic illustration of the flowchart outlining the results of the search strategy is shown in Figure 1.

To ensure that all possible publications were captured, multiple iterations of the search were processed. Boolean operators were routinely used to combine search terms, and advanced field tags were incorporated to refine the selection process in an attempt to limit the analysis to clinically oriented papers focused on healthcare. Reference lists from included articles were cross-checked to identify additional articles. Review articles and papers presented as conference proceedings were excluded. Articles published from January 2014 to January 2024 with full text available on PubMed and restricted to the English language and human subjects were included. The full bibliographies of identified articles were reviewed and irrelevant studies including those focused exclusively on the waiting time while physically in the office were selectively removed. Where individual patients were included in multiple published series, the most complete or recent article was cited. Core themes focused on healthcare access were subjectively devised based on the review of the relevant peer-reviewed literature. An interpretive synthesis of the available publications was then presented focused on presenting the evidence evaluating digital disparities in healthcare.

## Results

### Search Results

The initial search yielded 511 articles. After screening of these articles on title and abstract, a total of 373 studies proceeded to full-text screening. Another 126 articles were excluded because they were review articles (*N* = 79), were designed as narratives or case reports (*N* = 20), were position papers (*N* = 11), used duplicative data (*N* = 9), or were abstracts only or conference proceedings (*N* = 7). A total of 247 original, peer-reviewed studies on digital disparities and/or inequities in healthcare thus were included and formed the basis for this systematic review. Among these 247 publications, a total of 78 (32%) were published between January 2014 to December 2018, and 169 (68%) were published between January 2019 and January 2024. The vast majority of these studies (191 of 247) originated from within North America with the remaining from Europe (*N* = 20), Asia (*N* = 19), the Middle East (*N* = 10), and Latin/South America (*N* = 7).

### Identified Themes

The 247 original, peer-reviewed studies we identified differed significantly in their research design, methods, and endpoints. All had a primary focus of presenting and elucidating disparities in the use of digital health technologies. While the variability in which results were reported across studies precluded direct comparisons and aggregation of data, disparities that were highlighted generally pertained to groups in lower income categories (*N* = 97), of underrepresented minority status (*N* = 50), of elderly age (*N* = 49), of more limited education (*N* = 49), who were unemployed (*N* = 47), living in rural or urban regions (*N* = 40), and of non-English language status (*N* = 11). Notably, nearly all studies identified in this review determined greater than 1 at-risk group.

**Figure 2.**
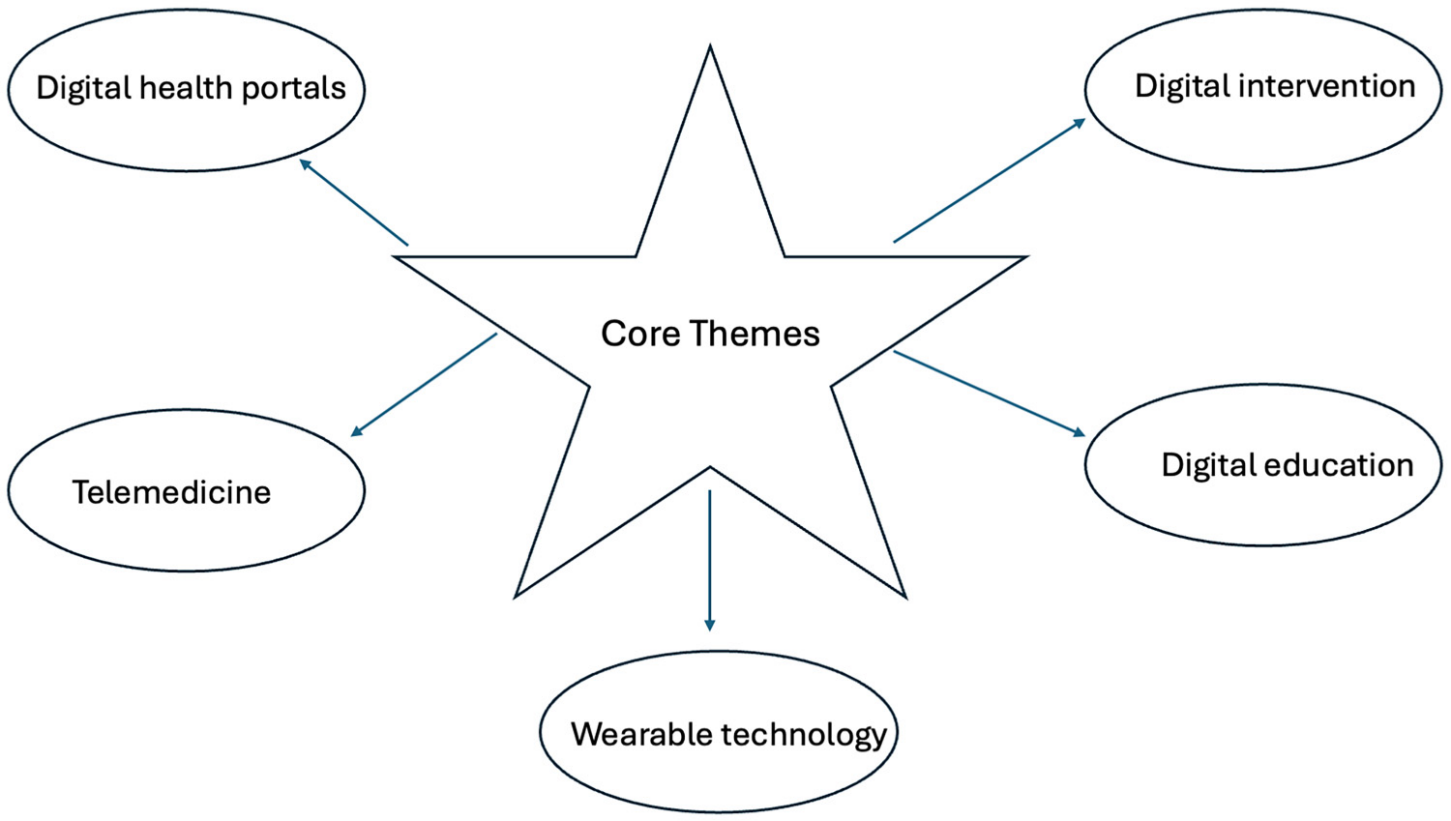
Core themes identified related to digital disparities in healthcare.

For the purpose of framework development, the core themes could broadly be categorized into those related to digital health portals (*N* = 74), telemedicine (*N* = 57), healthcare wearables (*N* = 49), digital intervention tools (*N* = 35), and virtual education (*N* = 32). Recognizing that overlap often existed in these themes, individual studies were stratified based on their primary focus. Table 1 provides a summary of the identified studies grouped into the themes that emerged. Artificial intelligence and/or machine learning was a component of 30 (12%) of the identified publications. Sixty-nine (28%) and 33 (13%) studies utilized multi-center databases and population-based registries, respectively. One hundred and ninety studies originated from academic medical centers (77%) with the remainder from community-based settings. The most common diseases analyzed, when specified, were related to general medicine/family practice (*N* = 42), surgical care (*N* = 29), mental health (*N* = 25), cardiovascular disease (*N* = 17), and cancer (*N* = 12). The remaining 122 studies (49%) did not specifically report on any specialization. The sample size of human subjects ranged from 744 to 120,550 (mean = 5503 patients). Figure 2 outlines the core themes that were identified through this systematic review of the evidence.

**Table 1.** Summary of Evidence-Based Literature on Digital Health Disparities.

Theme	Number of studies	Platforms
Digital health portals	74	Patient messaging; electronic medical records; test results; appointment scheduling; prescription requests and refills; provider questions
Telemedicine	57	Consultation and follow up visits; care coordination; referral intake; nurse check-ins; mental health counseling
Healthcare wearables	49	Disease monitoring; vital sign biosensors; preventive medicine; physical activity tracking; medication reminder alerts
Digital intervention tools	35	Behavioral health prompts; chat bot and artificial intelligence-based health assistance; physical therapy regimens
Virtual education	32	Asynchronous learning; self-assessment modules; health libraries; symptom surveys; provider ratings; payor and hospital information

## Discussion

**Figure 3.**
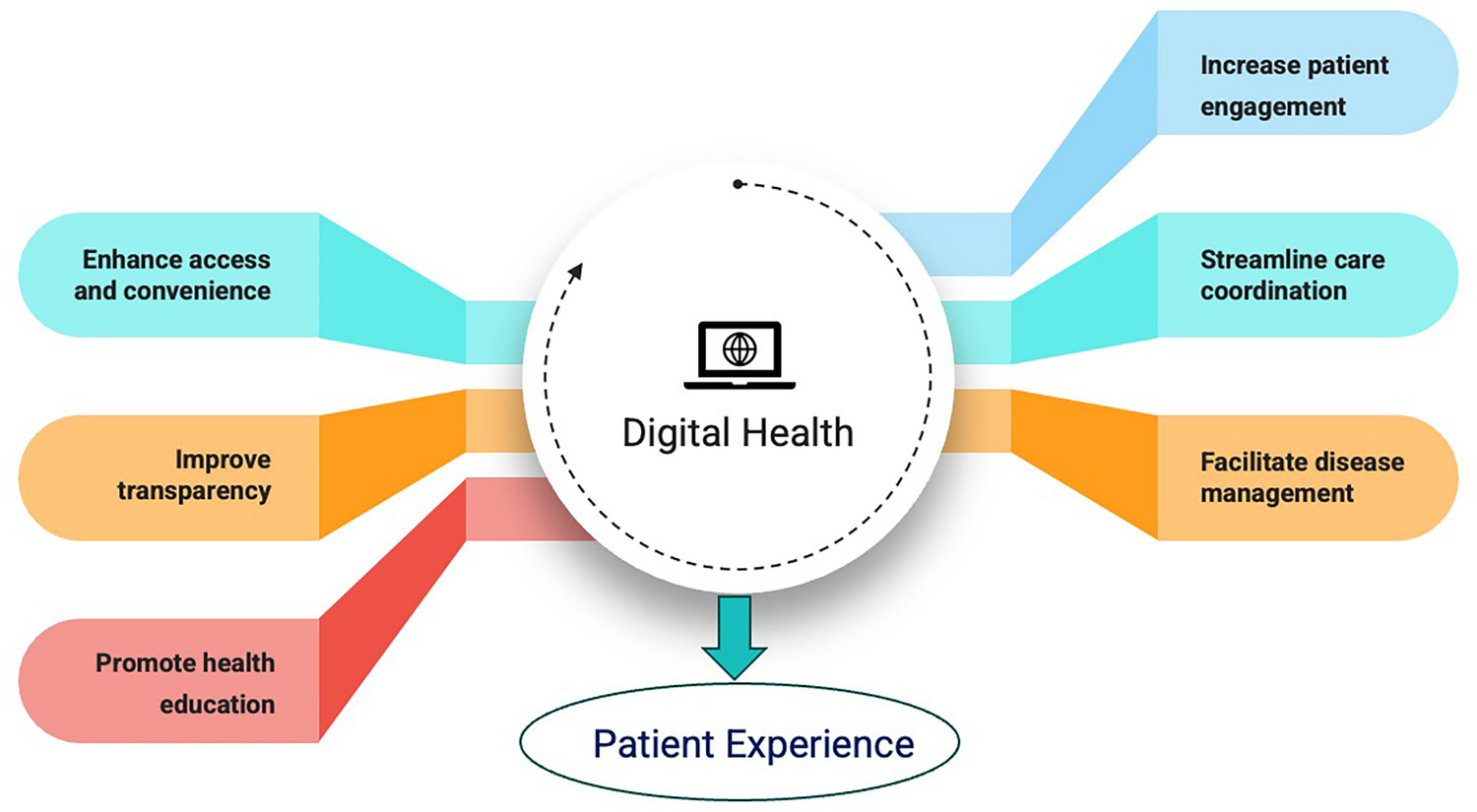
Relationship between digital health and patient experience.

The advent of digital health platforms has fundamentally transformed delivery paradigms in modern medicine and continues to shape the provider-patient relationship. At the heart of digital health is its potential to enhance the patient experience through increased personalization (Figure 3). While the merits of digital technology in ushering in an exciting new era promoting an unprecedented degree of patient-centric care, its potential to drive a larger gap between the “haves” and the “have-nots” should raise concern.

As demonstrated in this review, digital health has introduced new disparities with respect to its acceptance, access, and utilization—across nearly every aspect of technology implementation. This is largely because levels of digital health literacy vary widely across the population. For instance, data from the Pew Research Center in 2023 showed that approximately 5% and 20% of the population in the United States do not use the internet and lack broadband services in the household, respectively.^4^ Given the increasing reliance on digital health as a means of disseminating health-related information, it is thus not surprising that digital inclusion has been recognized as a new super determinant of health.^5^ In this respect, a notable theme of this review was the consistent identification of a variety of vulnerable segments—including patients from underrepresented backgrounds, lower socioeconomic status, rural communities, and decreased educational levels—who are at risk for falling behind the “digital divide.” Given the rapid incorporation of technological advances in healthcare, ensuring that digital solutions to address practical problems are integrated across all segments of the population must be prioritized.

In many ways, digital health has revolutionized patient-centric care. For instance, by offering users the ability to communicate with their provider teams at their convenience, digital patient portals, secure internet-based platforms directly linked to an electronic medical record, have been proposed as a powerful way of promoting patient engagement and shared decision-making. Given that information exchanges can be handled in the comfort of a patient's own home, these digital portals naturally facilitate a more user-friendly approach to access. However, the evidence clearly shows that utilization of digital portals varies widely across society and tend to be disproportionately accessed by segments viewed as privileged.

Tuan et al analyzed digital health utilization among 102,342 patients at 18 family medicine clinics considering such functionalities as messaging, health information management, billing, and education.^6^ The investigators found that patients living in rural areas as well as those from underrepresented minority groups were significantly less likely to use the messaging features. In addition, patients from areas with higher graduation rates from both high school and college were more likely to use the messaging domain. Significant differences in usage were also observed based on insurance status. In another study, Yoon et al analyzed 536 patients with multiple chronic conditions and showed on multivariate analysis that lower portal activity was significantly associated with inadequate health literacy, older age, female sex, and minority race.^7^ Griffin et al similarly conducted a multi-center study of 28,942 cancer patients and identified male sex, minority racial status, rural dwelling, not working, and limited broadband access with lower odds of portal access.^8^

Telemedicine appointments have also become more accepted in recent years. Notably, the coronavirus pandemic not only led to the widespread adoption of telemedicine, specifically video-only encounters, but also generated plentiful data starkly showing a concerning trend of disproportionate utilization across society.^9–11^ Indeed, the worsening of health disparities related to unequal use of telemedicine has been referred to as the “tragical paradoxical effect.”^12^ For instance, Adepoju et al analyzed patterns of telemedicine utilization in primary care across federally qualified health centers and showed that African Americans and Latinos were 35% and 51%, respectively, less likely to use virtual care compared to Whites.^13^ Iasiello et al. also analyzed 20,953 distinct patient visits across a regional health system and showed that African Americans and those lacking internet access were significantly less likely to participate in telehealth visits.^14^ On multivariate analysis, African American patients were 40% less likely to use telemedicine compared to White patients. Qian et al similarly showed that Latinos, Asians, Spanish-speaking, low-income, and Medicaid patients were significantly less likely to have used telemedicine after an analysis of 29,421 encounters among cancer patients.^15^ Notably, in a study of 955,352 primary care visits, Hsueh et al identified limited English proficiency as associated with decreased utilization of telemedicine.^16^

Disparities have also been prominently documented in the utilization of wearable healthcare devices which have utility in monitoring conditions, promoting patient engagement, and/or reporting alerting symptoms.^17^ For instance, studies have consistently shown that use of continuous glucose monitoring among diabetics is significantly lower among vulnerable segments of the population.^18–20^ Foster et al analyzed the compliance rates of 22,697 patients and showed that continuous glucose monitoring was lower among African Americans compared to Whites across all age ranges and income levels.^19^ Furthermore, Kanbour et al. showed that African Americans were significantly less likely to discuss continuous glucose monitoring with their providers compared to non-African Americans.^20^ With respect to cardiovascular disease, Chandrasekaran et al evaluated potential associations between the use of wearable healthcare devices and socio-economic factors using data from a national survey.^21^ The investigators showed a correlation between increased utilization and high-income levels; additionally, African American and Latino patients were 17% and 15%, respectively, less likely to use wearable devices. Another study from the US Health Information National Trends Survey showed that among the general population, younger subjects, those in higher income brackets, Whites, and those with higher levels of technology self-efficacy were more likely to use wearable healthcare devices.^22^ When logistic regression analysis was performed to identify potentials associations between socioeconomic factors and the willingness to share wearable device data with providers, the only two variables that were significant were race and marital status. Interestingly, White adults were three times more likely compared to non-Whites, and married individuals were half as likely compared to single adults to share their wearable data with providers. In another cohort study using wearable device usage data collected from 10,414 participants across the Adolescent Brain and Cognitive Development Study, total device wear time during the observation was notably shorter among African American children compared to White children.^23^

Digital interventions through mobile technology have also been shown to be subjected to disparities. While the popularity of mobile technology to promote health behavior and to manage chronic disease has soared, it is increasingly evident that utilization of navigational tools or virtual assistants is uneven across society. Mitchell et al analyzed data among the elderly population from the technology module of the Health and Retirement study and showed that compared to whites, African Americans and Latinos were less likely to use technology for health-related purposes even after accounting for demographic characteristics, education, and health conditions.^24^ Indeed, patients from diverse, low-income communities have consistently used mobile health technology at lower rates than the rest of society.^25–27^ For these at-risk patients, the opportunity to gain health education through digital means is often lost as well. For example, Fareed et al analyzed data from the Health Information National Trends Survey and showed that cancer survivors who reported not using digital platforms as a source of health education were more likely to be older, reside in rural areas, have less education, and of underrepresented minority status.^28^

While the published studies have consistently shown that patients from historically disadvantaged groups are less likely to use digital health tools, few have evaluated the underlying reasons explaining this phenomenon. Sakar et al suggested that cultural sensitivity might be lacking with the implementation of digital technologies and identified 3 themes which might negate usage from a series of interviews and direct observations in a diverse population: lack of confidence with technology, frustration with design features and navigation, and interest in having technology to support their self-management.^29^ Irizarry et al showed that the negative sentiments regarding this technology typically related to potential difficulty troubleshooting without having access to live technical support and feeling pressured to adopt new communication methods that were viewed as unnecessary compared to traditional ones.^30^ Moreover, hesitation also stemmed from concerns regarding the user-friendliness of the technology coupled with the lack of training or experience with the use of computers. Hoogenbosch et al similarly suggested that the discrepancy between effort expectancy and performance expectancy as contributing to the acceptance of digital health strategies.^31^ The relationship between socioeconomic factors, particularly income and education, on accessing mobile health has been documented.^32–35^

The role of mistrust is achieving equity with respect to digital health needs to be explored.^36–38^ Research has demonstrated that minority patients, when compared with Whites, frequently report higher rates of dissatisfaction with their relationships with physicians, poorer communication with their providers, and poorer overall patient experience.^39^ Given this cultural landscape, it is likely that issues related to implicit bias affect digital health utilization. Indeed, in an analysis of a nationally representative survey data, Richwine et al showed that African American and Latino patients were offered the usage of digital portals at significantly lower rates than Whites.^40^ More recently, Loeb et al reported the results of a randomized clinical trial evaluating the role of medical trust in acceptance of online health information regarding prostate cancer screening and clinical trials.^41^ The investigators showed that African American individuals were subjectively more trustful of online or digital content when it was delivered by a racially concordant presenter.

Potential interventions to address digital disparities largely require considering these in the larger framework of social determinants and structural inequities. To illustrate, the term “digital redlining” was introduced to describe inequities in access to technology infrastructure, including access to health care, education, employment, and social services.^42^ While efforts to promote education, awareness, and health literacy as a means of improving inclusivity have been described, it is likely more systematic approaches will be necessary to bridge the divide.^43–45^ Targeted initiatives under the Center for Medicare and Medicaid delivery system, as well as the Centers for Disease Control and Prevention, are increasingly considering social determinants, and it is important that digital literacy be integrated.^46,47^ Indeed, Smith et al presented an 18-point “Digital Universal Precautions” as a mandate for healthcare organizations as a pro-active means of addressing health literacy.^48^ To start, the development and implementation of screening initiatives to identify patients who are at risk for being left behind the “digital divide” should be prioritized considering the influence of socioeconomic factors including those related to education, geography, and income. On a more granular basis, it must also be recognized that effectively screening for digital disparities in healthcare should focus on digital literacy, internet access, technology comfort, and device ownership— with the goal of identifying gaps in usage so that interventions could be tailored to address these disparities. Given that the spread of misinformation online can also pose a challenge to digital health literacy, the need for training so that individuals can reliably distinguish credible sources from unreliable ones is also apparent. In this sense, pro-active engagement on the part of health systems will be crucial to help the population as a whole navigate the increasing amount of digital information accessible to the masses.

Lastly, initiatives to promote diversification of the workforce and to address implicit bias may also improve trust among the underserved and promote acceptance of digital technologies. Along these lines, ensuring that digital tools are culturally competent may also promote their use. For example, wearables have been shown to often lack accuracy in ethnic minorities, and algorithms to optimize their applicability in these populations are being recognized.^49^ Ultimately, efforts to view digital technologies through a variety of cultural lenses may provide a more robust framework so that access and utilization is more even across society.^50^

### Limitations

Due to the nature of this work and the heterogenous works identified for inclusion, considerable discretion was utilized to select the articles included and to categorize them into the theme-based groups. As a result, some degree of overlap existed between the defined core themes which was centered on the issue of digital disparities. Thus, this study did not present any quantitative data but instead only offered an interpretive synthesis of the available literature in qualitative fashion. As such, one of the primary limitations of this study was that the presented framework to consider the many facets of digital disparities was largely devised as an interpretive construct of the author. Since all of the studies reviewed were retrospective in nature and generally originating from single-institutional experiences, unforeseen biases could have confounded the findings espoused by this review.

It is also acknowledged that the subject of digital health disparities is one that is highly complex regarding its sheer breadth and scope. Thus, the aim of this study was not to present a comprehensive view of this topic but to provide a practical introduction to readers so that they can better appreciate the magnitude of how digital disparities could impact the patient experience in a real-world setting.

## Conclusions

Digital technologies have ushered in a new era in healthcare in which patients have unprecedented access to information like never before. While the goal of these digital platforms is to prioritize engagement and shared decision-making in healthcare, the effectiveness of such systems in reaching all patients equally across the population must be questioned. Indeed, it is abundantly clear that certain segments of society are disproportionately less likely to use and gain from these emerging technologies and are at risk for being further marginalized in the future. Given the breadth and depth of disparities that were identified—across nearly every aspect of digital health—the potential for increasing health inequities with the introduction of such technologies must be acknowledged. As the link between digital health and patient experience becomes better established, it is crucial that health organizations look to narrow these inequities with respect to access and utilization. Some strategies that were identified in this review include those focused on screening for digital literacy; education; workforce development; and community-based engagement. How stakeholders, including health systems and legislative bodies alike, opt to invest in strategies to address these disparities could mean the difference between enabling widespread access across society or leaving subpopulations unevenly behind the “digital divide.”
